# Nanoenhancer for improving naked DNA electrotransfection *In vivo*

**DOI:** 10.3389/fbioe.2023.1181795

**Published:** 2023-05-09

**Authors:** Yifei Wang, Chunxi Wang, Justin Sylvers, Tatiana Segura, Fan Yuan

**Affiliations:** Department of Biomedical Engineering, Duke University, Durham, NC, United States

**Keywords:** nanoenhancers, nanoparticles, naked DNA delivery, electrotransfection, electroporation

## Abstract

**Introduction:** Electrotransfection (ET) is a non-viral approach widely used for delivery of naked nucleic acids. Its efficiency can be increased *in vitro* by treatment of cells with various small molecule enhancers. However, these enhancers often fail to improve ET *in vivo*, presumably due to rapid clearance in tissues after local injection, reducing their cellular uptake. To this end, we propose to develop a new type of ET enhancers, which we term nanoenhancer, that diffuse slowly in tissues and are poorly absorbed by blood and lymph microvessels.

**Methods:** Two nanoenhancers were synthesized with alginate (Alg) and chitosan (Chi) with or without poly (ethylene imine) (PEI). They were used to treat cells *in vitro* or mouse muscle in the hind leg *in vivo* prior to ET of plasmid DNA coding reporter genes. At 24 hours post ET, the efficiency of ET was quantified, and compared with that in the untreated controls. Changes in lysosomal size and acidity post nanoenhancer treatment were measured with fluorescence microscopy techniques.

**Results and discussion:** We observed that the pretreatment of cells with the nanoenhancers could enhance the ET efficiency and cell viability in both C2C12 and HCT116 cells *in vitro*, and the nanoenhancer pretreatment had similar effects on the ET efficiency *in vivo*. Mechanisms of the enhancement were related to transient inactivation of lysosomal functions triggered by the nanoenhancer treatment. The concept of nanoenhancer will lead to development of new enhancers that can be used to improve ET efficiency *in vivo*, highlighting its potential in clinical applications.

## 1 Introduction

Electric pulses have been widely used to facilitate transfection of naked plasmid DNA (pDNA) into cells for transgene expression (Sardesai and Weiner, 2011; Young and Dean, 2015; Cervia et al., 2017; Mao et al., 2020; Wang et al., 2021). The electro-transfection (ET) method is simple, cost-effective, versatile, and safe. Thus, it has been used in various applications, such as cancer gene therapy, vaccination against viral infection, and cell engineering (Tamura and Sakata, 2003; Sardesai and Weiner, 2011; Young and Dean, 2015; Roth et al., 2018). To enhance the efficiency of ET, scientists have identified small molecule enhancers, such as histone deacetylases inhibitors (Vaughan et al., 2008; Badding and Dean, 2013) and certain ingredients in commercial ET buffers (e.g., those for Nucleofection^®^) (Hu and Li, 2014; Sherba et al., 2020), that can facilitate pDNA transport or cargo gene expression in targeted cells. In a previous study, we discovered that treatment of cells with a non-reducing sugar (NRS), such as sucrose, trehalose, and raffinose, could reduce naked pDNA degradation in lysosomes (Mao et al., 2020), a common problem for non-viral gene delivery. As a result, the treatment can substantially increase the ET efficiency in cell lines and primary cells *in vitro* (Mao, 2019; Mao et al., 2020). Mechanism of the increase is two-fold. First, the NRS treatment leads to formation of large (>500 nm), nonacidic vesicles, called amphisome-like bodies (ALBs). They hinder transport of pDNA-carrying vesicles to lysosomes. Second, the treatment enlarges lysosomes, resulting in pH increase in lysosomes that in turn causes inactivation of lysosomal enzymes. Both the ALB formation and the lysosome enlargement are likely to be due to the NRS molecules intercalating the phospholipid head groups via hydrogen bonds because the same phenomenon has also been observed in the study of phospholipid vesicles in aqueous solutions (Roy et al., 2016).

Though the NRS treatment improves ET efficiency *in vitro*, we observed that it failed to improve ET in mouse muscle and solid tumor tissues (data not shown). To our knowledge, few small molecule enhancers have been used successfully to improve ET *in vivo*. We hypothesize that the failure is due to rapid clearance of these molecules, reducing their cellular uptake. The hypothesis is based on the fact that small, hydrophilic molecules have relatively short half-lives (e.g., a few minutes) in tissues, due to rapid diffusion to surrounding tissues and absorption by blood and lymph microvessels (Dang et al., 1994; Strasser et al., 1995; Mahoney and Saltzman, 1999; Truskey et al., 2009). Therefore, we proposed to develop a new type of ET enhancer, called nanoenhancer, which are much larger than small molecules. Our hypothesis is that the large size allows the new enhancers to stay in tissues for a longer period (e.g., a few days) (Yuan et al., 1994; Mahoney and Saltzman, 1999; Wong et al., 2011; Kadam et al., 2012; Hoshyar et al., 2016). To test the hypothesis, we searched macromolecules and nanoparticles (NPs) in the literature that could induce formation of large vesicles in cells, similar to the ALBs and enlarged lysosomes observed in NRS treated cells (Mao et al., 2020). A finding in the search was the NPs synthesized with cationic polymers that have a large buffering capacity. They can raise osmotic pressure in the vesicles post endocytosis, causing vesicle enlargement and membrane rupture (Akinc et al., 2005; Benjaminsen et al., 2013; Routkevitch et al., 2020). The phenomenon is explained by the well-known “proton sponge” hypothesis. Although it differs from the mechanism of the vesicle enlargement caused by the NRS treatment, we considered that the NPs with cationic polymers have a potential for enhancing the ET efficiency, especially *in vivo*. In this proof-of-concept study, the NPs were synthesized with alginate and chitosan with or without polyethyleneimine (PEI) (You et al., 2006; Benjaminsen et al., 2013; Cervia et al., 2017; Routkevitch et al., 2020). The goal of the study is to demonstrate that treatment of cells with the nanoenhancers can enhance ET efficiency even in situations where the optimal pulsing parameters are used for the transfection, and that the nanoenhancers are incapable of pDNA transfection when used alone without pulsing.

## 2 Materials and methods

### 2.1 Chemicals and pDNAs

Sodium alginate (Alg, low viscosity), chitosan (Chi, 75%–85% deacetylated, MW 50–190 kDa), branched polyethyleneimine (PEI, MW 25 kDa), sodium hydroxide solution (NaOH, 1N), acetic acid (ACS reagent), and Amicon ultra-centrifugal filter (4mL, 100 kDa cutoff) were purchased from Sigma-Aldrich. Calcium chloride (dihydrate) were purchased from Mallinckrodt Chemicals. Propidium Iodide (PI, Invitrogen), hydrochloric acid solution (HCl, 1N), and standard RC dialysis membrane (Spectrum^®^ Laboratories, MWCO 3.5 kDa) were purchased through Thermo Fisher Scientific. D-Luciferin was purchased from Xenogen. The plasmid DNA (pDNA), pEGFP-N1, encoding enhanced green fluorescence protein (EGFP), were obtained from Clontech (#6085-1); pcDNA3-luciferase (#18964) encoding Firefly Luciferase gene and pDNA encoding Lysosomal-Associated Membrane Protein 1 (LAMP1)-mCherry (#45147) were purchased from Addgene.

### 2.2 Cell culture

The study used 2 cell lines, human colon cancer (HCT116) and mouse myoblast (C2C12) obtained from Duke University Cell Culture Facility (CCF). HCT116 cells were cultured in McCoy’s medium (Gibco), supplemented with 10% bovine calf serum (BCS, Avantor Seradigm) and 1% penicillin-streptomycin (Pen-Strep, Thermo Fisher Scientific). C2C12 cells were cultured in Dulbecco’s modified Eagle’s medium (DMEM, Gibco), supplemented with 10% BCS and 1% Pen-Strep as well. Cells were incubated in a humidified incubator at 37 °C in 5% CO_2_, passaged every 2–3 days.

### 2.3 Nanoparticle synthesis

The protocols for alginate-chitosan (Alg-Chi) or alginate-chitosan-polyethyleneimine (Alg-Chi-PEI) nanoparticle syntheses were adapted from previous studies for generation of Alg-Chi or alginate-polylysine nanoparticles (Rajaonarivony et al., 1993; De and Robinson, 2003; Bhunchu et al., 2016). The molecular weight of PEI was chosen to be 25 kDa, based on the design reported in previous studies that balances the trade-off between effective drug delivery and low cytotoxicity (Sadeghpour et al., 2018; Zhang et al., 2018). Briefly, a sodium alginate solution (0.2% w/v) was prepared in deionized water (DI H_2_O), with adjustment of pH to 4.9–5.0 by 1N HCl. A PEI solution (0.1% w/v) was prepared similarly in DI H_2_O, with pH adjusted to 5.5 using HCl. A chitosan solution (0.1% w/v) was prepared by dissolving chitosan powder in 1% v/v acetic acid solution, with pH adjusted to 4.6 using 1% w/v NaOH solution. The PEI and chitosan solutions might be mixed at the 1:1 ratio. Thus, the final concentrations of both PEI and chitosan in the mixture became 0.05% w/v. A CaCl_2_ solution was prepared in DI H_2_O as well, with a final concentration of 0.2% w/v. To prepare propidium iodide (PI)-loaded nanoparticles, PI (0.04 mM) was pre-mixed with the alginate solution under magnetic stirring at the speed of 1,200 rpm using stirring plate (Thermofisher Cimarec+™ series) for 10 min.

**FIGURE 1:**
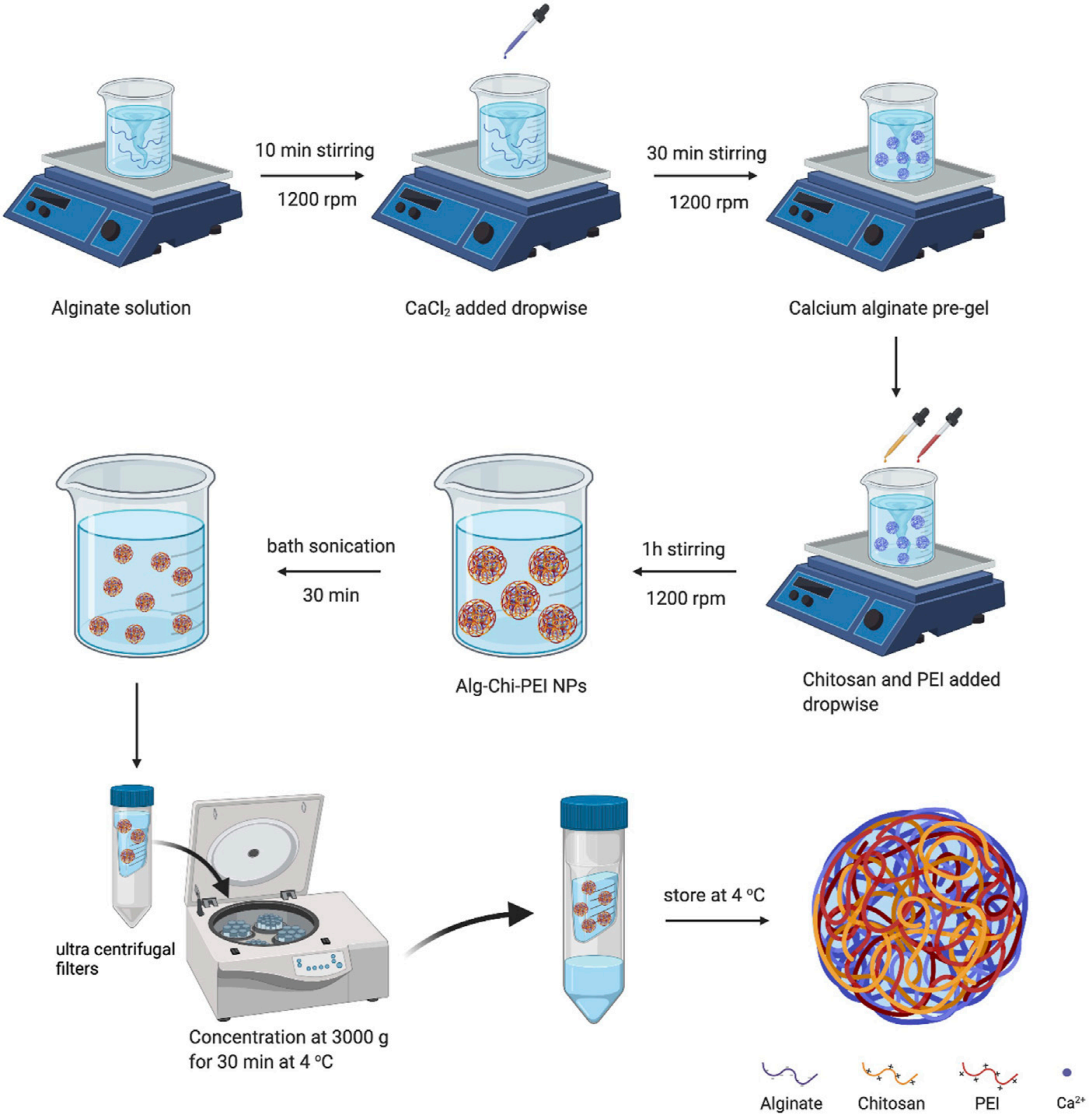
Schematic illustration of alginate-chitosan-PEI nanoparticle synthesis. The details of the synthesis are described in the Material and Methods section. Alg, Alginate; Chi, chitosan (Created with BioRender.com).

For the nanoparticle synthesis (see Figure 1), the CaCl_2_ solution was added dropwise into the alginate solution with or without PI under continuous magnetic stirring for 30 min at 1,200 rpm. Afterwards, the cationic polymer (chitosan or premixed chitosan and PEI) solution was gradually added dropwise into the stirring solution over an hour, with the mass ratio between total polycations and polyanions being 0.01, 0.05, 0.1 or 0.5. The nanoparticle suspensions were sonicated at 40 kHz frequency and 110 W power in a bath sonicator maintained at 37°C for 30 min. Then, the nanoparticles were purified and concentrated using ultra-centrifugal unit filtration at 3,000 g and 4°C.

### 2.4 Characterization of nanoparticles

The nanoparticle samples were fixed on TEM grids for 10 min, followed by washing with ultra-pure water to remove excess samples. The sample was then negatively stained using uranyl acetate for 5 min and visualized under a transmission electron microscope (FEI Tecnai G^2^ Twin). The average hydrodynamic diameter and zeta potential of nanoparticles in pure water were measured with the dynamic light scattering (DLS) technique in Omega cuvettes at room temperature (Litesizer 500, Anton Paar). All samples were evaluated in triplicates.

### 2.5 Cellular uptake of nanoparticles

Cells were seeded in 24-well plates at 0.5-1 x 10^5^ cells per well for 24 h. Then, the free PI or the PI-loaded nanoparticle sample was mixed with cell culture medium; and the cells were incubated in the new medium at 37°C in the CO_2_ incubator. One hour later, the cells were gently rinsed twice with PBS. Finally, the full culture medium was added to each well for imaging using a fluorescence microscope (Axio Vert A1 inverted microscope, Carl Zeiss).

### 2.6 Electrotransfection

The cells were seeded in 6-well plates at 0.5 × 10^6^ cells per well for 24 h. Then, they were incubated in the medium supplemented with a nanoparticle (10 μg/μL) for a certain period described in figure legends. After the incubation, the cultured cells at 70%–80% confluency were collected by 0.25% Trypsin-EDTA (Gibco). 10^6^ cells per sample were washed with PBS, and resuspended in 100 μL pulsing buffer, Opti-MEM GlutaMAX (Gibco), containing 1 μg pDNA. The sample was transferred to a disposable aluminum electroporation cuvette with 2-mm or 4-mm gap (Bio-Rad), and pulsed using the BTX ECM830 Square Wave Electroporation System (Harvard Apparatus). HCT116 cells were pulsed at a condition of 650 V/2 mm, 400-μs duration, 1 pulse; and C2C12 cells were pulsed at a condition of 250 V/4 mm, 10-ms duration, 2 pulses with 10 s interval. After pulsing, 1 mL of fresh full medium pre-warmed at 37°C was added to the cuvette, and the cells were cultured in 6-well plates overnight before flow cytometry analysis.

### 2.7 Flow cytometry analysis

The flow cytometry analysis was performed to quantify the electrotransfection (ET) efficiency and cell viability. Prior to the analysis, PI was added to the cell samples (1 μM) to stain dead cells, and the samples were fully vortexed. EGFP and PI signals were simultaneously detected in 488 nm and 633 nm channels, respectively, of a flow cytometer (NovoCyte, Agilent). The data were used to quantify the ET efficiency and cell viability as described in previous studies (Mao et al., 2020). Briefly, electrotransfection effectiveness (eTE) was defined as the percentage of live cells expressing EGFP (PI-/EGFP+). Expression level was defined as geometric mean of EGFP fluorescence intensity per cell among PI-/EGFP+ cells. Similar to most previous studies (Haberl et al., 2013; Chang et al., 2016; Cervia, 2017; Mao et al., 2020), the cell viability was defined as the ratio of live cell numbers between experimental and control samples. In the current study, a live cell meant its plasma membrane to be impermeable to PI, i.e., PI-; and the cell viability (%) was calculated as 100 times the live cell ratio. Apparent Expression Level was defined as the product of eTE, expression level, and cell viability, which is a measure of the overall expression level in a sample.

### 2.8 Lysosome analysis in nanoparticle treated cells

C2C12 cells were electrotransfected with plasmid encoding LAMP1-mCherry (1 μg per 10^6^ cells) at 24 h prior to the Alg-Chi-PEI nanoparticle treatment (10 μg/μL). Then, the cells were incubated with the nanoparticle for 24 h, followed by staining with Lysotracker Green (LTG) and Hoechst (Invitrogen). The nanoparticle treated cells and untreated control cells were imaged under an Andor Dragonfly spinning disk confocal microscope equipped with a ×63 oil objective. Super-resolution imaging was performed using the super-resolution radial fluctuations (SRRF) method implemented in the Andor Dragonfly system. Colocalization of signals between red mCherry and green LTG in the images were determined with MATLAB^®^. The sizes (i.e., the two-dimensional projection area) and the numbers of LAMP1^+^ and LTG^+^ vesicles in randomly selected cells were determined with CellProfiler. The data were used to calculate the mean and the SEM of vesicle size, and the vesicle number per cell in each group.

### 2.9 Mouse study *in vivo*

CD1 mice (Charles River Labs) were used in the study based on a protocol adapted from the literature (Sokołowska and Błachnio-Zabielska, 2019). On Day 1, fur on both hind legs was shaved. Then, we injected 30 μL of 1xPBS into gastrocnemius muscle of the left hind leg (the internal control), and 30 μL of Alg-Chi-PEI nanoparticle in 1x PBS (20 μg/μL) into the same muscle of the right hind leg. On Day 2, the same muscle regions in right and left hind legs were injected with 25 μL of pDNA encoding luciferase (10 μg) in 1xPBS, followed by percutaneous application of electrical pulses to the muscle region of each leg through two stainless steel plate electrodes (10 × 10 mm) (BTX). A conductive gel was applied on electrodes before pulsing to ensure sufficient contact of the electrodes to the leg skin. The pulsing condition was 70 V/4 mm, 8 pulses, 100 ms each at 1 Hz frequency. On Day 3, the mice were injected intraperitoneally with D-luciferin at 24 h post electrotransfection. After 5 min, bioluminance images of mice were acquired with IVIS, and the radiance was measured for determination of luciferase expression level.

## 3 Results and discussion

### 3.1 Synthesis and characterization of nanoenhancers

We synthesized the alginate-chitosan-PEI NP by following the procedures shown in Figure 1. Briefly, CaCl_2_ solution was added drop-by-drop into alginate solution at room temperature to create a pre-gel state necessary for the ionic interactions in the next step between polyanions (alginate) and polycations (chitosan and PEI). Under high-speed stirring, positively charged chitosan and PEI in solutions were added dropwise into the pre-gel mixture to generate the polyelectrolyte complexes between oppositely charged polymers (Nakamura et al., 2020). The resulting solution was bath-sonicated to eliminate large complexes for improving the size homogeneity. The NPs were purified and concentrated by ultra-centrifugal unit filtration. For comparison, we also synthesized the alginate-chitosan NPs using a similar method (see the Material and Methods section).

**FIGURE 2:**
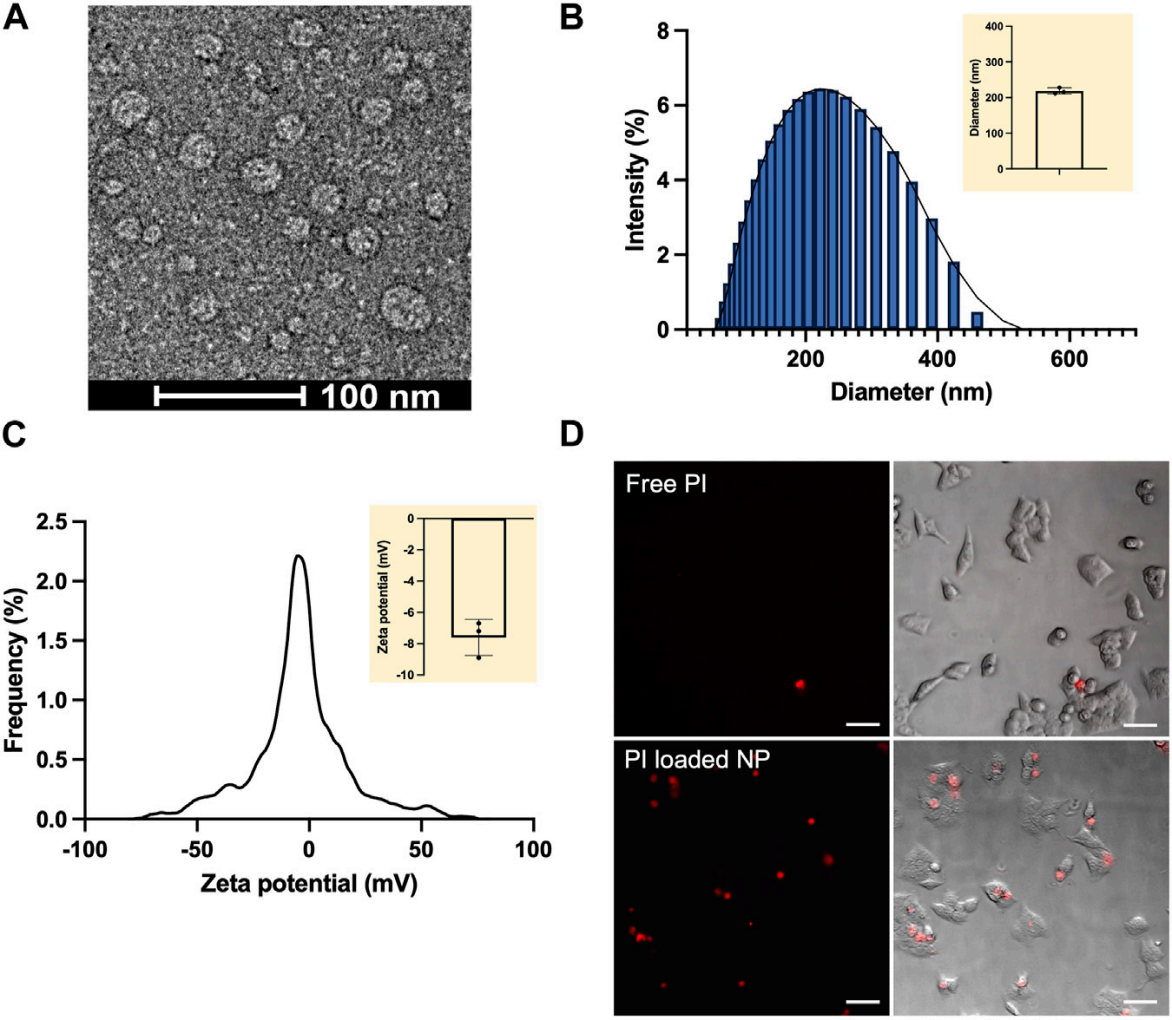
Characterization of Alg-Chi-PEI NPs. **(A)** Typical image of the NPs under transmission electron microscope (TEM). **(B)** Representative distribution of NP diameters measured with dynamic light scattering (DLS). Mean ± SD of the parameter from three independent tests shown in the inset were: 218.4 ± 7.1 nm. **(C)** Representative distribution of NP zeta potentials. Mean ± SD of the parameter from three independent tests shown in the inset were: −7.6 ± 1.2 mV. **(D)** Cellular uptake of PI. Free PI and PI-loaded NP solutions were used to treat HCT116 cells for 1 h. The cells were rinsed twice with PBS prior to the image acquisition. Equivalent PI concentrations in both solutions were 0.04 mM. Scale Bar: 50 μm.

The NPs were analyzed to determine their physicochemical properties. Under transmission electron microscope (TEM), the alginate-chitosan-PEI NPs appeared to be spherical with the diameter of 20–50 nm (Figure 2A). The size was consistent with the observation of similar nanoparticles consisted of chitosan and alginate (Li et al., 2008). It is well known that the mass ratio of different polymers in the formulation could influence the final size, stability, and hydrophilicity of NPs (Chen et al., 2017). In the current study, the stability of the NPs decreased with increasing the total polycations to polyanions (alginate) mass ratio (data not shown). When the mass ratio was higher than 0.5, the NPs started to precipitate as aggregates in the aqueous buffer. Stable NP formulation was achieved at the mass ratio of 0.01–0.05, with a hydrodynamic size of 200–250 nm measured with the dynamic light scattering (DLS) method. With the consideration of size and stability, we chose the mass ratio to be 0.01 in the final formulation, at which the hydrodynamic size of the NPs (mean ± SD) was 218.4 ± 7.1 nm (Figure 2B), and the zeta potential (mean ± SD) was −7.6 ± 1.2 mV measured with the electrophoretic light scattering method (Figure 2C). For the alginate-chitosan NPs, the hydrodynamic size (mean ± SD) was 205.0 ± 25.3 nm (Supplementary Figure S1A), and the zeta potential (mean ± SD) was −18.2 ± 7.7 mV (Supplementary Figure S1B), which were consistent to the data in the literature (Li et al., 2008; Niculescu and Grumezescu, 2022).

### 3.2 Cellular uptake of nanoenhancers

In addition to the size and charge, we evaluated cellular uptake of the alginate-chitosan-PEI NPs loaded with a red fluorescent dye, propidium iodide (PI). The PI-loaded NPs were slightly smaller than the empty NPs, with a mean hydrodynamic diameter of 167 nm. PI is commonly used for assessing cell viability because it cannot diffuse into cells with an intact plasma membrane. In the current study, the dye was used to demonstrate the capability of the NPs to enter the cells. HCT116 cells were incubated in the solution of free PI or PI-loaded NPs for 1 hour. Then, the cells were washed with PBS and visualized under a fluorescence microscope equipped with the Texas Red filter (Figure 2D). The red PI signal was observable in a tiny fraction of the cells incubated in the free PI solution, but many cells incubated in the PI-loaded NP solution, demonstrating that the alginate-chitosan-PEI NPs could be effectively internalized by the cells.

### 3.3 Application of nanoenhancer to enhance ET *in vitro*

**FIGURE 3:**
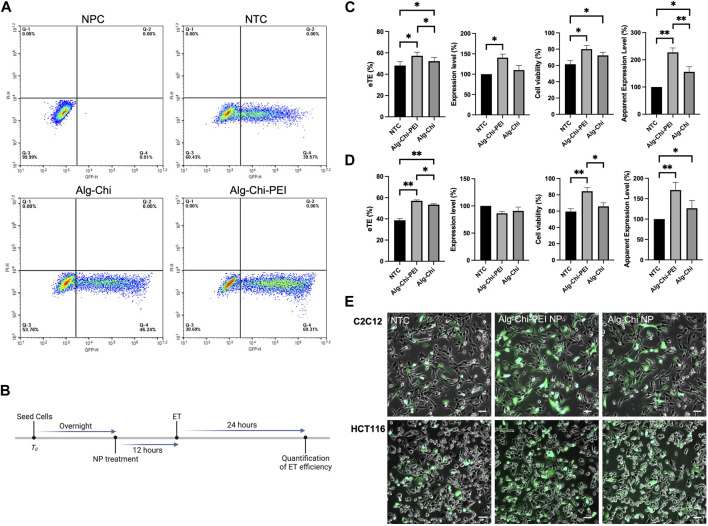
Effects of Alg-Chi-PEI nanoenhancer treatment on pDNA electrotransfection *in vitro*. **(A)** Typical flow cytometry data used for ET efficiency quantification. The plot shows data of C2C12 cells in non-pulsed control (NPC), non-treated control (NTC), and two NP treated groups, respectively. **(B)** Timeline of the *in vitro* experiment. Cells were treated with the nanoenhancer (10 μg/μL, 12 h) prior to electrotransfection of pDNA encoding EGFP. Cells in the matched control groups were not treated, i.e., NTC. The eTE, expression level, cell viability, and apparent expression level were determined at 24 h post ET. **(C)** and **(D)** ET efficiencies and cell viabilities for C2C12 cells and HCT116 cells, respectively. The data in treated groups were normalized by those in matched NTC groups. Error bars, SEM. **(C)** *n* = 7; *p < 0.05, **p < 0.01, Wilcoxon test. **(D)** *n* = 5; *p < 0.05, **p < 0.01, Mann-Whitney test. **(E)** Typical overlays of bright-field and fluorescence images showing EGFP expression in transfected cells at 24 h post ET. Scale Bar: 200 μm.

To determine if the NPs could be used to enhance pDNA electrotransfection *in vitro*, we treated the cells with the Alg-Chi-PEI NPs in complete medium for different periods prior to electrotransfection of a plasmid encoding EGFP (pEGFP). Effects of the treatment on ET efficiency were quantitatively evaluated with flow cytometry at 24 h post ET (Figure 3A), using four parameters: i) electrotransfection effectiveness (eTE), percent of viable cells expressing the EGFP protein; ii) expression level, geometric mean of fluorescence intensity per cell among viable, EGFP positive cells; iii) cell viability, percent of viable cells; and iv) apparent expression level, the product of eTE, expression level, and cell viability; it is a measure of the average gene expression level per cell in a group. Our data showed that the optimal pretreatment period, which led to the highest ET efficiency, was 12 h (Supplementary Figure S2). This period was chosen in all subsequent experiments *in vitro* (Figure 3B).

The pretreatment of cells with the Alg-Chi-PEI NP could enhance ET efficiency and cell viability in both C2C12 and HCT 116 cells *in vitro* (Figures 3C, D). The overall enhancements in term of the apparent expression level were 120% and 70% for C2C12 and HCT116 cells, respectively, compared with the non-treated controls. In a negative control experiment, we exposed the Alg-Chi-PEI NP pretreated cells to the pEGFP in the ET buffer but did not deliver any electric pulses to these cells. After 24 h, there was little EGFP expression in these cells (data not shown), indicating that the NP treatment alone was insufficient to transfect pDNA into cells. The quantitative data were consistent to the qualitative observation of the cells at 24 h post ET (Figure 3E), in which the treated cells displayed more, brighter green fluorescence signals than the cells in non-treated controls. In a similar experiment, we observed that the pretreatment of cells with the Alg-Chi NP could also enhance ET efficiency and cell viability, although the enhancement levels were less, compared to the data in the Alg-Chi-PEI NP treated groups (Figure 3). The difference in the ET efficiency, caused by the incorporation of PEI in the NPs, might be due to a high amine content in PEI that could induce a robust proton sponge effect after the NPs being endocytosed. As a result, lysosomes were more enlarged, leading to an increase in lysosomal pH and the deactivation of nucleases involved in pDNA degradation.

### 3.4 Mechanisms of enhancement in cells

**FIGURE 4:**
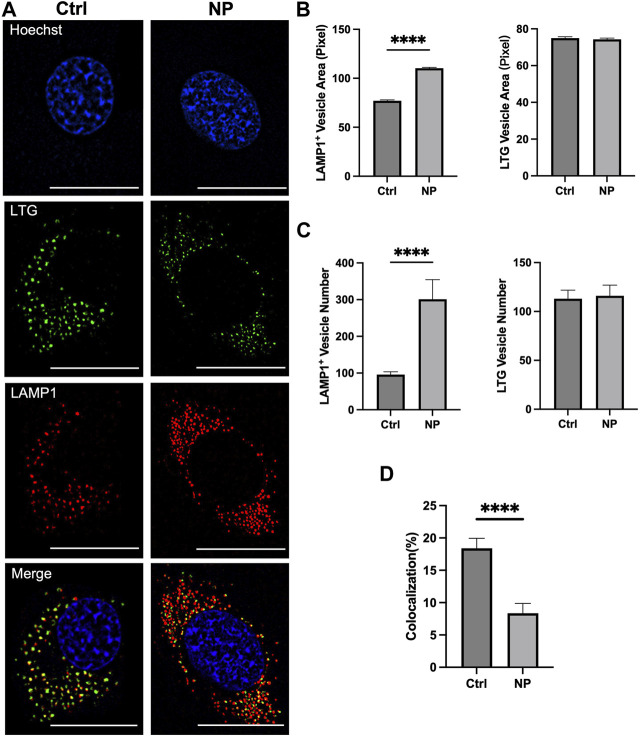
Effects of Alg-Chi-PEI nanoenhancer treatment on lysosomal size and pH. **(A)** C2C12 cells expressing LAMP1-mCherry were treated with Alg-Chi-PEI nanoenhancer for 24 h, followed by staining with Lysotracker Green (LTG) and Hoechst dyes. Scale Bar: 20 μm. **(B)** Quantification of projection area of LAMP1^+^ and LTG^+^ vesicles. The area per pixel is 0.00277 µm^2^. **(C)** Quantification of LAMP1^+^ and LTG^+^ vesicle numbers per cell. **(D)** Quantification of colocalization coefficient. It was calculated as the percent of LAMP1 signal co-localized with the LTG signal. In all plots, bars and error bars denote mean and SEM, respectively; n = 50; ****p < 0.0001, Mann-Whitney test.

Next, we investigated mechanisms of the ET improvement in nanoenhancer treated cells. The investigation was focused on whether the nanoenhancer could increase the lysosomal pH since the cationic amine groups in the nanoenhancers can be protonated in acidic environment (Akinc et al., 2005; Benjaminsen et al., 2013; Routkevitch et al., 2020). The study used LAMP1-mCherry-expressing cells treated with lysotracker green (LTG), where the LAMP1-mCherry is a red fluorescent marker for lysosomes and the LTG is a marker for acidic vesicles. The fluorescence images showed that the nanoenhancer treatment increased the size and the number of LAMP1 positive vesicles, and reduced the number of yellow pixels, an indicator of colocalization between LAMP1-mCherry and LTG (Figure 4A). Quantitatively, the nanoenhancer treatment increased the size of lysosomes by 43% (Figure 4B), lysosome number by 214% (Figure 4C), and reduced the colocalization coefficient by approximately 50% (Figure 4D). Meanwhile, the same treatment had little effects on the size and the number of acidic vesicles (Figures 4B, C). These data demonstrated that less lysosomes were acidic following the nanoenhancer treatment. Since the acidic environment is required for the activity of lysosomal enzymes (Isaac et al., 2000), our data suggests that nuclease activities are significantly inhibited in cells treated with the Alg-Chi-PEI nanoenhancer, which enhances the ET efficiency by reducing pDNA degradation.

### 3.5 Application of nanoenhancer to enhance ET *in vivo*

**FIGURE 5:**
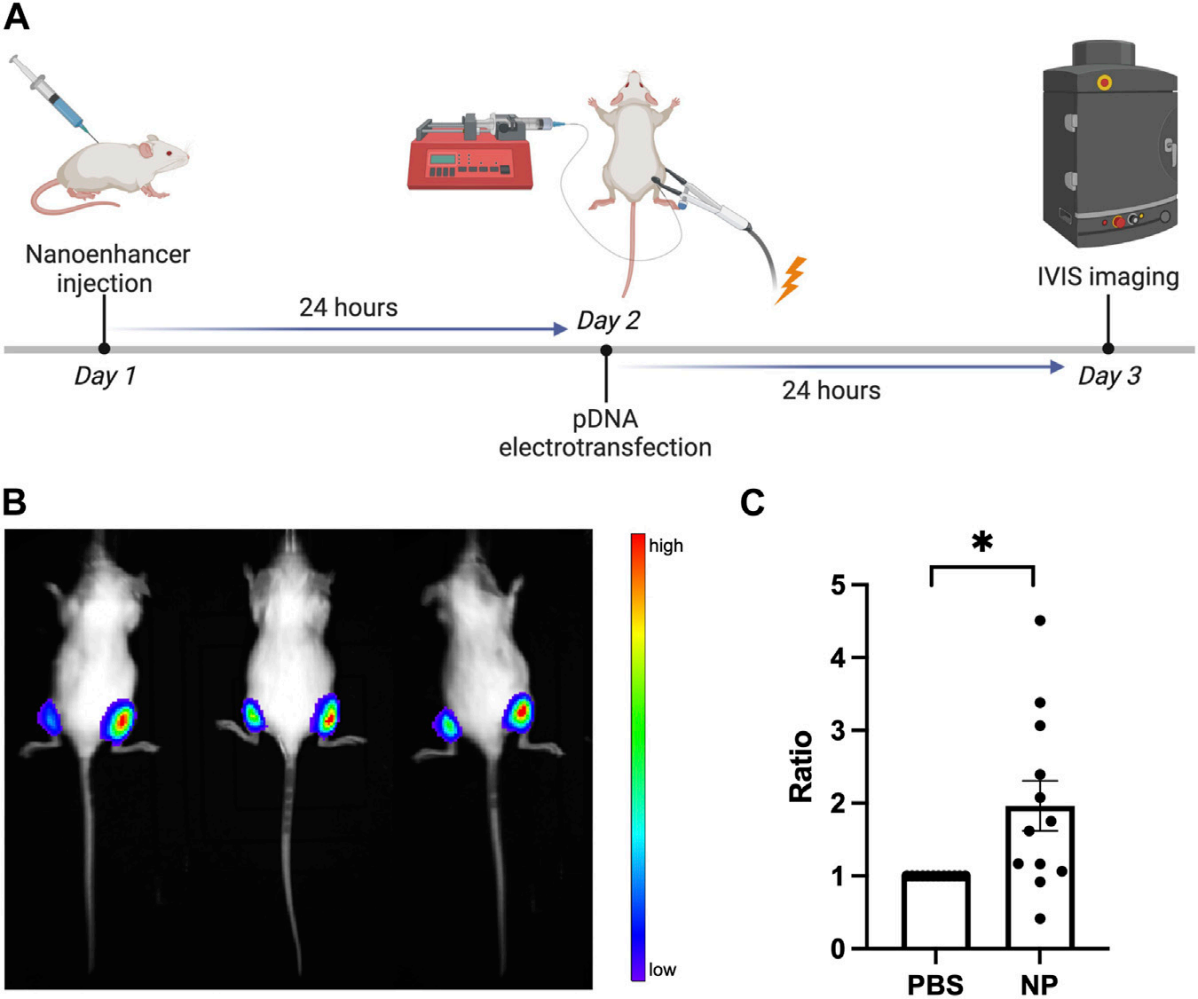
Effects of Alg-Chi-PEI nanoenhancer treatment on pDNA electrotransfection *in vivo*. **(A)** Timeline of *in vivo* ET experiment. The mouse muscles (gastrocnemius) in the right and left hind legs were treated with the nanoenhancer (20 μg/μL, 24 h) and PBS (i.e., the internal control), respectively, prior to electrotransfection of pDNA encoding luciferase. At 24 h post ET, the bioluminescence signal was measured with the IVIS. **(B)** Typical image of the bioluminescence in mice, and **(C)** quantitative measurement of the signal intensity. For each animal, the radiance data from the right leg was normalized by that from the left leg. Error bar, SEM; *n* = 12; *p < 0.05, Wilcoxon test.

In addition to the enhancement *in vitro*, we investigated effects of the Alg-Chi-PEI pretreatment on pDNA electrotransfection *in vivo*. The experimental design is shown in Figure 5A. The nanoenhancer was injected into the mouse muscle in the right hind leg, and as an internal control, PBS was injected into the left leg. The electrotransfection of pDNA encoding luciferase was performed in both legs. The luciferase expression level was evaluated by measuring the bioluminescence radiance in both control (left) and treated (right) muscles using an *in vivo* imaging system (IVIS^®^ Spectrum). The data showed that the Alg-Chi-PEI treatment approximately doubled the gene expression level, compared to the PBS control (Figures 5B, C), which was quantitatively consistent with the apparent expression level *in vitro* (Figure 3C). These data together indicated that the nanoenhancer treatment had similar effects on ET in cultured cells and animals.

## 4 Conclusion

We propose a new strategy to chemically enhance electrotransfection of pDNA *in vivo*. It is achieved through pretreatment of tissues with nanoenhancers. In this proof-of-concept study, we demonstrate that the nanoenhancer treatment could improve pDNA electrotransfection with minimal cytotoxicity both *in vitro* and *in vivo*, highlighting its promise as a universal ET enhancer. To our knowledge, this is the first study showing that nanoenhancers can be used to improve ET *in vivo*. In future studies, we will expand the strategy to develop nanoenhancers through encapsulation of the small molecule enhancers in nanoparticles. The nanoenhancers have a potential to improve various applications of ET *in vivo* including those in the clinic.

## Data Availability

The original contributions presented in the study are included in the article/supplementary material, further inquiries can be directed to the corresponding author.
