# Supplementary material for: Nanoenhancer for improving naked DNA electrotransfection In vivo
Source: Front Bioeng Biotechnol. 2023 May 9;11:1181795. doi: 10.3389/fbioe.2023.1181795 (PMC10203387; doi:10.3389/fbioe.2023.1181795)
Supplement: Supplementary file 1 [file DataSheet1.pdf]

# *Supplementary Material*

# Nanoenhancer for Improving Naked DNA Electrotransfection In Vivo

**Yifei Wang, Chunxi Wang, Justin Sylvers, Tatiana Segura, Fan Yuan***

*** Correspondence:** Fan Yuan: fyuan@duke.edu

## Supplementary Figures

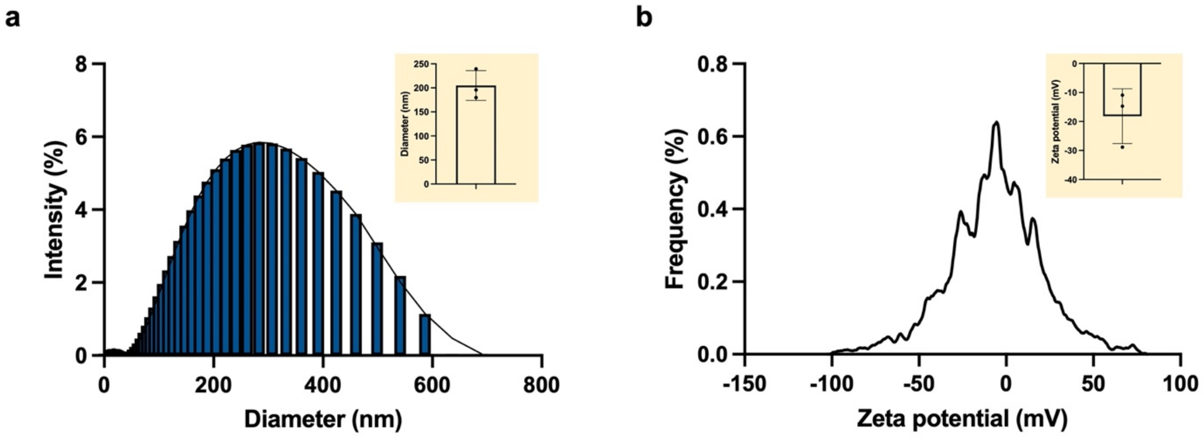


**Figure S1.** Characterization of Alg-Chi nanoparticles. **a)** Representative Distribution of their diameters measured by dynamic light scattering (DLS). Mean±SD of the parameter from three independent tests shown in the inset were: 205.0±25.3 nm. **b)** Representative Distribution of their zeta potentials. Mean±SD of the parameter from three independent tests shown in the inset were: –18.2±7.7 mV.

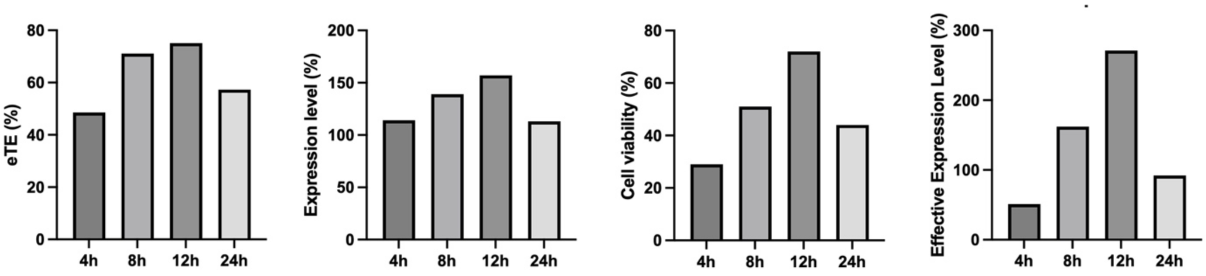


**Figure S2.** Effects of Alg-Chi-PEI treatment period on ET efficiency in C2C12 cells. The protocols for the treatment and the electrotransfection were the same as those described in the legend of Figure

3, except that the treatment period was varied from 4 to 24 hours. The data suggested the optimal period to be 12 hours.
